# Marginal Notes, July 2024. Taken for Granted

**DOI:** 10.1099/jmm.0.001876

**Published:** 2024-08-20

**Authors:** Timothy J. J. Inglis

**Affiliations:** 1 Schools of Medicine and Biomedical Sciences, The University of Western Australia, Crawley, Western Australia, WA 6009, Australia

**Keywords:** blood culture, blood culture bottles, bloodstream infection, diagnostic stewardship, sepsis, supply chain

In July 2024, the American Food and Drug Administration added blood culture bottles to the Medical Devices Shortage List [1], due to an emerging shortage of bottles from one of the main biotech suppliers. Shortly afterwards, the Centres for Disease Control and Prevention issued a health advisory on how to cope with a blood culture bottle supply shortage [2]. On this side of the globe, where all blood culture bottles are imported, the supply problem already threatened an imminent loss of significant capability. Plans were made, measures introduced and additional monitoring was set up. At the time of writing, there is still no end in sight.

The dire consequences of running out of blood culture bottles were considered as long ago as 2002, in the context of white powder incidents and related domestic bioterrorism concerns [3]. Recognition of the vulnerability of health supply chains prompted the drafting of a set of plans to triage blood cultures to conserve stocks of blood culture bottles in times of crisis [4]. Now that we are faced with exactly this problem, questions are being asked about how long bottles can be used after the manufacturer’s expiration when the recommendation is to avoid any use after expiry. Locally, the advice is no more than a month after expiry, even though the bacterial growth-supporting properties of standard blood culture media may not deteriorate for a much longer period [5]. The lack of data on exactly how long before blood culture media expires is just one indication of how much we take blood culture for granted. So far, Australia appears to be the only place where a 1-month extension of the manufacturer’s expiration date has been allowed.

A moderated conversation with the blood culture bottle supplier revealed that the supply problems were more complex than first thought and had led to the reactivation of the manufacture of the previously used glass blood culture bottles [6]. Recovery of bacteria using another supplier’s plastic (polycarbonate) and glass bottles found comparable yield and time to detection of positive cultures in glass and plastic bottles [7]. The argument for the switch to plastic lies in improved staff safety and lighter weight. Microbiological non-inferiority is assumed for a wider range of species, culture media variants and other plastic formulations. Reversion to glass blood culture bottles is thus a backward step, but one that should not adversely affect overall culture yield or efficiency.

Blood culture is a commonly used procedure that provides a surprisingly low return on effort and is prone to both false positives (contamination) and false negatives (failure to grow due to antimicrobial action, poor culture collection timing or fastidious species). Now seems like a good time to ask how to improve the return on blood culture effort [4]. How can the number of blood culture bottles used be reduced without losing critical laboratory results? Recommendations in the CDC advisory highlight a reduction in unnecessary duplication and avoiding contamination during specimen collection [2]. Adding a little detail, this diagnostic stewardship favours dropping the so-called ‘routine’ blood culture collected as a check of treatment efficacy not postponing overnight blood culture collection until the routine morning phlebotomy round and eliminating the collection of low-yield multiple blood culture sets.

There is more to good stewardship of scarce resources than parsimonious use of blood culture bottles. If there was greater clarity on the reason for media expiry, improved stock control [7] and the application of artificial intelligence to fine-tune the initial decision to collect [8], wasteful use of blood culture bottles could be reduced and the effort could be targeted more effectively to the patients in greatest need of clinical decision support.

TJJ Inglis, WA, 31 July 2024.

## References

[1] Medical Devicexs Shortages List, Food and Drugs Administration. https://www.fda.gov/medical-devices/medical-device-supply-chain-and-shortages/medical-device-shortages-list.

[2] (2024). Disruptions in availability of Becton Dickinson (BD) BACTEC™ Blood Culture Bottles Blood Culture Bottles. CDC Health Alert Network, July 23, 2024, 2:45 PM ET. CDCHAN-00512. https://emergency.cdc.gov/han/2024/han00512.asp.

[3] Shapiro DS (2003). Surge capacity for response to bioterrorism in hospital clinical microbiology laboratories. J Clin Microbiol.

[4] Fabre V, Carroll KC, Cosgrove SE (2022). Blood culture utilization in the hospital setting: a call for diagnostic stewardship. J Clin Microbiol.

[5] Hardy L, Vermoesen T, Gleeson B, Ferreyra C, Dailey P (2024). Blood culture bottles remain efficient months after their expiration date: implications for low- and middle-income countries. Clin Microbiol Infect.

[6] CDC’s Laboratory Outreach Communication System (LOCS). https://www.cdc.gov/locs/calls/archive/2024/July.html.

[7] Kazzazi F, Kazzazi D, Kukendra-Rajah K, Basarab M (2021). What can we learn from supermarkets? an application of the Poisson distribution (order-up-to) model to improve blood culture bottle supplies. Future Healthc J.

[8] McFadden BR, Inglis TJJ, Reynolds M (2023). Machine learning pipeline for blood culture outcome prediction using sysmex XN-2000 blood sample results in Western Australia. BMC Infect Dis.

